# Outcome of COVID-19 patients treated with VV-ECMO in Tyrol during the pandemic

**DOI:** 10.1007/s00508-023-02301-5

**Published:** 2023-11-10

**Authors:** Andreas Peer, Fabian Perschinka, Georg Lehner, Timo Mayerhöfer, Peter Mair, Juliane Kilo, Robert Breitkopf, Dietmar Fries, Michael Joannidis

**Affiliations:** 1 Division of Intensive Care and Emergency Medicine, Department of Internal Medicine, Medical University Innsbruck, Anichstr. 35, 6020 Innsbruck, Austria; 2 Department of Anaesthesiology and Intensive Care Medicine, Medical University Innsbruck, Innsbruck, Austria; 3 Department of Cardiac Surgery, Medical University of Innsbruck, Innsbruck, Austria

**Keywords:** Newly initiated center, Experienced center, RRT delay, Complications, Age

## Abstract

**Introduction:**

A small percentage of patients infected with the severe acute respiratory syndrome coronavirus 2 (SARS-CoV‑2) showed severe respiratory deterioration requiring treatment with extracorporeal membrane oxygenation (ECMO). During the pandemic surges availability of ECMO devices was limited and resources had to be used wisely. The aim of this analysis was to determine the incidence and outcome of venovenous (VV) ECMO patients in Tyrol, when criteria based on the Extracorporeal Life Support Organization (ELSO) guidelines for VV-ECMO initiation were established.

**Methods:**

This is a secondary analysis of the Tyrol-CoV-ICU-Reg, which includes all patients admitted to an intensive care unit (ICU) during the coronavirus disease 2019 (COVID-19) pandemic in Tyrol. Of the 13 participating departments, VV-ECMO was performed at 4 units at the University Hospital Innsbruck.

**Results:**

Overall, 37 (3.4%) of 1101 patients were treated with VV-ECMO during their ICU stay. The hospital mortality rate was approximately 40% (*n* = 15). Multiorgan failure due to sepsis was the most common cause of death. No significant difference in survival rates between newly initiated and experienced centers was observed. The median survival time of nonsurvivors was 27 days (interquartile range, IQR: 22–36 days) after initiation of VV-ECMO. Acute kidney injury meeting the Kidney Disease: Improving Global Outcomes (KDIGO) criteria occurred in 48.6%. Renal replacement therapy (RRT) was initiated in 12 (32.4%) patients after a median of 18 days (IQR: 1–26 days) after VV-ECMO start. The median length of ICU and hospital stays were 38 days (IQR: 30–55 days) and 50 days (IQR: 37–83 days), respectively.

**Discussion:**

Despite a rapidly increased demand and the resulting requirement to initiate an additional ECMO center, we could demonstrate that a structured approach with interdisciplinary collaboration resulted in favorable survival rates similar to multinational reports.

**Supplementary Information:**

The online version of this article (10.1007/s00508-023-02301-5) contains supplementary material, which is available to authorized users.

## Introduction

During the years 2020–2022 intensive care units (ICU) were faced with the challenge of treating critically ill patients infected with severe acute respiratory syndrome coronavirus 2 (SARS-CoV-2). These patients were mostly admitted to ICUs due to respiratory failure [1] requiring noninvasive ventilation (NIV) or invasive mechanical ventilation (IMV) [2, 3]; however, in a small percentage of patients the use of venovenous extracorporeal membrane oxygenation (VV-ECMO) was inevitable due to progressive respiratory deterioration [4]. Due to the high demand many centers previously not providing ECMO services started to do so during the pandemic. As the number ECMO devices are limited and performing extracorporeal life support (ECLS) treatment requires substantial material and staffing resources, the implementation of criteria to avoid a first come first serve approach and to achieve the best possible outcome for the patients was necessary. Despite the application of the criteria, the outcome of patients remains uncertain being influenced by various complications such as bleeding [5] and additional organ failures, such as acute kidney injury (AKI) [6]. Additionally, various other factors depending on physicians’ decisions, such as the time point of cannulation, settings of mechanical ventilation and weaning procedures reportedly impact outcome [7, 8]. Therefore, a center’s experience in performing ECLS, which is reflected in the number of treatments performed per year, is a quality parameter and associated with the mortality rate [9, 10]. During the pandemic, many ICUs established the use of ECMO treatment, but outcomes showed huge variations between centers as well as countries. Furthermore, a tendency to increased mortality associated with increased ECMO use was reported [11, 12].

To provide a basis for more homogeneous indications for ECMO, criteria published by an expert panel based on the Extracorporeal Life Support Organization (ELSO) guidelines for initiating VV-ECMO were established (ESM table 1) [13–15]; however, finally the initiation remained an individualized decision made by the physician in consultation with an interdisciplinary team.

The aim of this analysis was to determine the incidence and outcome of COVID-19 patients treated with VV-ECMO in Tyrol, Austria, where provision of ECMO was restricted to one tertiary hospital (four different units) and, thus, resources were very limited. Consequently, strict criteria for VV-ECMO initiation had to be established from the very beginning of the pandemic.

## Methods

This is a secondary analysis of the Tyrol-CoV-ICU-Reg, a prospective multicenter registry, including data from 13 different ICUs allocated in 8 hospitals (list of all participating ICUs available in ESM table 2) in the period from 1 February 2020 until 14 December 2022. Of the ICUs five were located at the University Hospital of Innsbruck, four units provided VV-ECMO treatment, one of which started for the first time during the pandemic. Inclusion criteria for the registry were admission to an ICU and a positive SARS-CoV‑2 PCR. The Tyrol-CoV-ICU-Reg has been published in detail previously [16]. This registry was approved by the local ethics committee (Nr. 1099/2020).

Initiation of VV-ECMO was standardized for all the centers supervised by one ECMO team on the basis of criteria published by an expert panel based on ELSO guidelines for initiating VV-ECMO [13]. The ECMO service was provided for the regions of Tyrol and Vorarlberg, two states in Austria comprising about 1.1 million inhabitants.

Data were collected until death or discharge from hospital, whichever occurred first. If patients were transferred from one ICU to another ICU (within participating ICUs), the stays were linked and analyzed as one stay.

Only adult patients (age ≥ 18 years) were included in this analysis. Baseline characteristics were extracted from the patient information system and recorded in the Tyrol-CoV-ICU-Reg. Based on the documentation of the intensive care units, interventions and their duration as well as medication and complications were collected. The sequential organ failure assessment (SOFA) score [17] and the simplified acute physiology score 3 (SAPS 3) were calculated at the time of ICU admission.

Considering respiratory support, invasive mechanical ventilation (IMV) and noninvasive mechanical ventilation (NIV) were distinguished. Ventilation was classified as IMV when it was performed via endotracheal intubation or tracheostoma, while NIV was categorized into nasal high flow and positive pressure ventilation (CPAP/ASB) conducted by a mask or helmet. An AKI was diagnosed by applying the Kidney Disease:Improving Global Outcomes (KDIGO) guidelines including increased serum creatinine or decreased urine output [18]. Continuous venovenous hemofiltration (CVVH), continuous venovenous hemodialysis (CVVHD) and continuous venovenous hemodiafiltration (CVVHDF) were summarized as renal replacement therapy (RRT). All interventions had to be performed for at least 2 h a day to be considered.

Comorbidities were obtained by searching the patient information system and were grouped into cardiovascular disease, hypertension, diabetes mellitus/prediabetes, renal comorbidity, hepatic comorbidity, neurological comorbidity, respiratory comorbidity, solid cancer, non-solid cancer, and immunosuppression.

Continuous variables are presented as median (interquartile range, IQR) while categorical variables are shown as numbers with corresponding percentage. Statistical analyses were performed with the software SPSS (version 27; IBM Corp., Armonk, NY, USA). Normal distribution was tested by Shapiro-Wilk tests. The significance of continuous variables was evaluated by conducting t‑tests and Mann-Whitney U‑tests, while the χ^2^-test was used for categorical variables. Correlations were calculated by applying the η coefficient and analysis of variance.

A two-sided *p* value < 0.05 was considered statistically significant.

All data were collected with an eCRF and REDCap electronic data capture, a web platform for managing databases and surveys created by Vanderbilt University and hosted by the Department of Medical Statistics, Information and Health Economics, Medical University Innsbruck [19, 20].

## Results

Overall, 1101 patients were included in the Tyrol-CoV-ICU-Reg during the defined period. The median age of all registered patients was 66 years (IQR: 55–75 years) and the majority (68.3%) were male. Of the patients 568 (51.8%) required invasive mechanical ventilation due to respiratory deterioration, of whom 37 (3.4%) patients were treated with VV-ECMO during the ICU stay, 21 patients were treated at a newly initiated center and 16 patients were treated at experienced centers. VV-ECMO patients were younger (53 years; IQR 47–58 years) and predominantly male (78.4%). Only one patient was fully vaccinated. Additional characteristics are presented in Table 1. Of 37 patients treated with VV-ECMO, 22 (59.5%) were discharged alive from hospital, while 15 (40.5%) died and 13 of these patients died during the ICU stay. In most nonsurvivors (8; 53.3%) sepsis accompanied by multiple organ failure was diagnosed as the cause of death. One patient died because of hemorrhagic complications. Four patients died after treatment was withdrawn due to a poor prognosis. Two patients died after discharge from the ICU, one from a hemorrhagic complication, the other one due to recurrent refractory respiratory failure. The difference in survival rates between newly initiated and experienced centers was not significantly different (66.7% vs. 50%; *p* = 0.306). After initiation of VV-ECMO the median survival time was 27 days (IQR: 22–36 days) in nonsurvivors. One patient was bridged to bilateral lung transplantation but did not survive to hospital discharge.

**Table 1:** Characteristics of COVID-19 patients treated with VV-ECMO in Tyrol

		Overall (*n* = 37)	Survivors (*n* = 22)	Nonsurvivors (*n* = 15)	*p*
Sex*	Male	29 (78.4%)	16 (72.7%)	13 (86.7%)	0.312
Age°(years)		53 (47–58)	50 (43–56)	56 (53–61)	0.026
Age group* (years)	< 40	5 (13.5%)	4 (18.2%)	1 (6.7%)	0.030
	40–60	23 (62.2%)	16 (72.7%)	7 (46.7%)	
	60–80	9 (24.3%)	2 (9.1%)	7 (46.7%)	
SOFA score°		7 (5–8)	6 (5–9)	7 (4–7)	0.772
SAPS III°		50 (47–60)	49 (46–58)	57 (49–63)	0.045
Fully vaccinated*		1 (2.7%)	1 (4.5%)	0	0.403
**Comorbidities**					
Number of comorbidities*		2 (0–2)	1 (0–2)	3 (1–4)	0.001
Cardiovascular*		4 (10.8%)	1 (4.5%)	3 (20.0%)	0.137
Hypertension*		15 (40.5%)	5 (22.7%)	10 (66.7%)	0.008
Diabetes mellitus*	No diabetes	29 (78.4%)	18 (81.8%)	11 (73.3%)	0.827
	Diabetes type 1	0	0	0	
	Diabetes type 2	6 (16.2%)	3 (13.6%)	3 (20.0%)	
	Prediabetes	2 (5.4%)	1 (4.5%)	1 (6.7%)	
Renal*		3 (8.1%)	0	3 (20.0%)	0.029
Liver*		3 (8.1%)	0	3 (20.0%)	0.029
Immunosuppression*		2 (5.4%)	1 (4.5%)	1 (6.7%)	0.779
COPD*		2 (5.4%)	0	2 (13.3%)	0.078
Asthma*		1 (2.7%)	0	1 (6.7%)	0.220
No comorbidities*		10 (27.0%)	9 (40.9%)	1 (6.7%)	0.021
**Interventions**					
IMV*		37 (100.0%)	22 (100.0%)	15 (100.0%)	–
NIV*		20 (54.1%)	12 (54.5%)	8 (53.3%)	0.942
NHF*		13 (35.1%)	7 (31.8%)	6 (40.0%)	0.609
Prone positioning*		36 (97.3%)	21 (95.5%)	15 (100.0%)	0.403
Vasopressors*		37 (100.0%)	22 (100.0%)	15 (100.0%)	–
RRT*		12 (32.4%)	3 (13.6%)	9 (60.0%)	0.003
Delay from ECMO to RRT initiation (days)°		18 (1–26)	13 (3–23)	18 (1–29)	1
Days IMV°		33 (26–53)	33 (24–53)	33 (27–54)	0.725
Days NIV°		2 (1–4)	2 (1–6)	2 (1–4)	0.970
Days NHF°		2 (2–4)	4 (1–7)	2 (2–3)	0.445
Days prone positioning°		9 (5–13)	9 (5–13)	9 (6–14)	0.849
Days RRT°		32 (9–38)	36 (34–37)	11 (9–39)	0.482
Days ECMO°		25 (14–30)	20 (11–29)	27 (20–35)	0.075
**Outcome**					
Acute kidney injury*	No AKI	19 (51.4%)	15 (68.2%)	4 (26.7%)	0.056
	KDIGO I	3 (8.1%)	2 (9.1%)	1 (6.7%)	
	KDIGO II	2 (5.4%)	1 (4.5%)	1 (6.7%)	
	KDIGO III	13 (35.1%)	4 (18.2%)	9 (60.0%)	
ICU mortality*		13 (35.1%)	0	13 (86.7%)	–
Hospital mortality*	Newly initiated centers	21 (56.8%)	14 (63.6%)	7 (46.7%)	0.306
	Experienced centers	16 (43.2%)	8 (36.4%)	8 (53.3%)	
Length of stay hospital° (days)		50 (37–83)	63 (42–104)	40 (34–65)	0.083
Length of stay ICU° (days)		38 (30–55)	38 (29–55)	35 (32–65)	0.867

*IMV* invasive mechanical ventilation, *NIV* noninvasive ventilation, *NHF* nasal high flow, *RRT* renal replacement therapy, *ECMO* extracorporeal membrane oxygenation, *AKI* acute kidney injury, *IQR* interquartile range, *KDIGO* Kidney Disease: Improving Global Outcomes, *ICU* intensive care unit

* number (%), ° median (IQR)

**Fig. 1:**
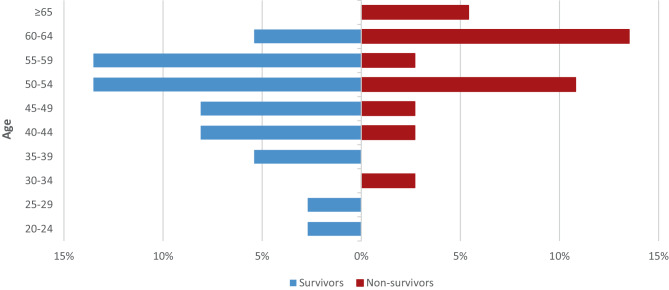
Age distribution (years) of VV-ECMO patients in Tyrol

Nonsurvivors were older and had a higher SAPS III score. A significant correlation between the age and ICU mortality (correlation coefficient: 0.327; *p* = 0.049) or hospital mortality (correlation coefficient: 0.369; *p* = 0.025) was found. A higher rate of nonsurvivors was found over 60 years old (Fig. 1).

In addition to IMV (all patients), prone positioning was conducted in the majority of the patients who survived (95%) and all the patients who died (100%; *p* = 0.403). No difference in the length of mechanical ventilation (*p* = 0.725) and prone positioning (*p* = 0.849) was seen; however, the duration of VV-ECMO treatment was longer in nonsurvivors as compared to survivors (27 days, IQR: 20–35 days vs. 20 days, IQR: 11–29 days, *p* = 0.075).

### Complications and outcome

Pulmonary embolism was detected in two survivors and five nonsurvivors. In the survivors a deep vein thrombosis was simultaneously diagnosed. Bleedings of at least type 2 according to the Bleeding Academic Research Consortium (BARC) [21] were observed in 8 (36.4%) survivors and 11 (73.3%) nonsurvivors (*p* = 0.027). One bleeding was fatal. The median number of administered red cells concentrates was 4 (IQR: 3–9.5) per patient, although in nonsurvivors significantly more concentrates were administered as compared to survivors (9 vs. 4; *p* = 0.020). In all nonsurvivors a co-infection was observed during the ICU stay (100% had bacterial co-infections, 73.3% had fungal co-infections), whereas two patients (9.1%) of the surviving group remained without co-infection (86.4% had bacterial co-infections, 86.4% had fungal co-infections) (Table 2).

**Table 2:** Interventions and complications during ICU stay

	Overall (*n* = 37)	Survivors (*n* = 22)	Nonsurvivors (*n* = 15)	*p*
*Disease-related medication*				
Antiviral drugs targeting SARS-CoV-2*	32 (86.5%)	19 (86.4%)	13 (86.7%)	0.979
Corticosteroids*	33 (89.2%)	19 (86.4%)	14 (93.3%)	0.503
Platelet inhibition*	7 (23.3%)	4 (23.5%)	3 (23.1%)	0.977
LMW heparin*	26 (83.9%)	16 (94.1%)	10 (71.4%)	0.087
*ECMO-related medication*				
Unfractionated heparin*	1 (3.2%)	1 (5.9%)	0	0.356
Direct oral anticoagulants*	4 (12.9%)	3 (17.6%)	1 (7.1%)	0.385
Argatroban*	15 (48.4%)	8 (47.1%)	7 (50.0%)	0.870
Red blood cell concentrates°	4 (3–10)	4 (2–5)	9 (4–15)	0.020
*Complications*				
Myocardial infarction*	0	0	0	–
Pulmonary embolism*	7 (22.6%)	2 (11.8%)	5 (35.7%)	0.112
Stroke*	0	0	0	–
Deep vein thrombosis*	2 (6.5%)	2 (11.8%)	0	0.185
Bleeding ≥ BARC type 2	19 (51.4%)	8 (36.4%)	11 (73.3%)	0.027
Other thromboembolism*	4 (12.9%)	2 (11.8%)	2 (14.3%)	0.835
Bacterial coinfection*	34 (91.9%)	19 (86.4%)	15 (100.0%)	0.136
Fungal coinfection*	30 (81.1%)	19 (86.4%)	11 (73.3%)	0.320
Viral coinfection*	11 (29.7%)	6 (27.3%)	5 (33.3%)	0.692

*SARS-CoV‑2* severe acute respiratory syndrome coronavirus type 2, *LMW* low molecular weight, *ECMO* extracorporeal membrane oxygenation, *BARC* Bleeding Academic Research Consortium

* number (%); ° median (IQR)

About half of the patients treated with VV-ECMO suffered from AKI (48.6%), with the rate being higher in nonsurvivors (73.3% vs. 31.8%; *p* = 0.056). Correspondingly, renal replacement therapy (RRT) was required more often in nonsurviving patients (60.0%) than in survivors (13.6%) (*p* = 0.003). RRT was initiated after a median delay of 18 days (IQR: 1–26 days) after VV-ECMO start. Only one patient required RRT before VV-ECMO.

Patients who survived were discharged from the ICU after 38 days (IQR: 29–55 days) and from hospital after 63 days (IQR: 42–104 days).

## Discussion

In this analysis, we report 37 COVID-19 patients treated with VV-ECMO in Tyrol, Austria, during the pandemic. Despite initiation of an additional center for performing VV-ECMO which ended up treating the majority of these patients, ICU and hospital mortality rates were similar or even better than those reported by meta-analyses performing ECMO in COVID-19 patients [22, 23]. The majority of patients who did not survive died from sepsis. Furthermore, nonsurvivors suffered from higher rates of AKI and required RRT more often.

The University Hospital Innsbruck was the only regional ECMO center providing service for an area of approximately 1.1 million inhabitants, which made it necessary to use resources wisely. Before the COVID-19 pandemic 5 ECMO devices were available. The number was increased to enable a maximum of 9 ECMO patients to be treated simultaneously while service for non-COVID-19 patients (e.g., cardiac surgery, trauma) still had to be guaranteed. In accordance with criteria based on ELSO guidelines, an attempt was made to select those patients who had the highest probability for survival. Age appears to be a major factor. An investigation already performed in the prepandemic era, showed significantly higher mortality in patients older than 65 years than in the control group [24]. Older age as a predictor of worse outcome on ECMO has been described in several studies during COVID-19 [25, 26]. The age distribution between nonsurviving ECMO patients and survivors was clearly different in our cohort and a significant correlation between age and hospital mortality could be established.

However, using age alone as the criterion for initiation does not seem to be useful. Besides the age, a variety of factors have been reported to be associated with patient outcome both at the time of ECMO initiation and during the course of treatment. For the time of initiation platelet count [25], PaO_2_/FiO_2_ ratio < 60 and pH < 7.2 have been shown [27] to influence the outcome.

After initiation, thrombotic events as well as bleeding are risk factors for poor outcome [28]. Our data confirm the significantly higher rate of blood transfusions in nonsurvivors; however, no conclusions can be drawn from our data concerning thrombotic events, probably due to the small sample size.

High rates of RRT have been reported for ECMO patients even before the pandemic [29]. In our cohort both AKI rates and especially RRT requirement were higher than usually reported for critically ill patients without COVID-19 [30] but similar to other reports investigating COVID-19 patients treated with ECMO. AKI can be triggered by the underlying disease complicated by lung-kidney interactions following respiratory failure and mechanical ventilation [31]; however, ECMO treatment may affect kidney function by several factors including hemolysis and microembolism [32]. In nonsurvivors a significantly higher rate of RRT was observed. In all patients RRT was started after ECMO initiation. This emphasizes the impact of AKI after ECMO initiation on patient outcome, which corresponds to other studies [33].

Despite the high demand, limited resources and the requirement to initiate a new ECMO unit which ended up treating the majority of COVID-19 patients, a favorable patient outcome similar to that of large meta-analyses was achieved [22]. Furthermore, comparing our results to those of similar healthcare systems even lower mortality rates can be reported [34]. Thus, our results demonstrate that similar survival rates can be achieved in newly initiated VV-ECMO centers compared to established VV-ECMO centers, if an interdisciplinary approach in collaboration with an experienced team is attempted.

In our study, once VV-ECMO treatment was initiated no significant difference in the treatment, such as the use of vasopressors between survivors and nonsurvivors was observed; however, the duration of VV-ECMO was shorter in survivors, which is consistent with the results from meta-analyses showing that reduced ECMO duration is significantly associated with lower risk for mortality [22]. Another meta-analysis showing longer ECMO duration and higher mortality in COVID-19 patients compared to influenza, discussed respiratory complications to be the reason for higher mortality [23]. With our available data we could not validate this finding.

### Strengths and limitations

Limitations of this analysis are its observational design and the relatively small number of patients. Strengths of this study are the multicenter approach and the comprehensive data collection for a defined region with its large catchment area and the uniform application of criteria for initiating ECMO treatment.

## Conclusion

Despite limited experience with VV-ECMO procedures prior to the COVID-19 pandemic, a structured approach regarding indication criteria and interdisciplinary collaboration accompanying the introduction of this technique in new ECMO centers may result in mortality rates similar to international standards and experienced VV-ECMO centers in the same catchment area.

### Supplementary Information

ESM tables 1 and 2
