# Supplementary material for: Outcome of COVID-19 patients treated with VV-ECMO in Tyrol during the pandemic
Source: Wien Klin Wochenschr. 2023 Nov 10;136(15-16):465–71. doi: 10.1007/s00508-023-02301-5 (PMC11327186; doi:10.1007/s00508-023-02301-5)
Supplement: Supplementary file 1 — ESM tables 1 and 2 [file 508_2023_2301_MOESM1_ESM.pdf]

Electronic supplemental material (ESM)

Outcome of COVID-19 patients treated with vv-ECMO in Tyrol during the pandemic

ESM table 1: Decision making for ECMO support in COVID-19 patients (13-15)

**Indications for vv-ECMO initiation**
PaO_2/FiO_2 <80mmHg for >6h*
PaO_2/FiO_2 <50mmHg for >3h*
pH <7.25 with $PaCO_2$ ≥60 mmHg for 6h*
** after optimization of respiratory treatment*

**Absolute contraindications**	**Relative contraindications**
Rejection by the patient	Age >65 years* (depending on the biological age)
Pre-existing severe neurological deficit, advanced dementia	Ventilation duration prior ECMO >7 days
End-stage disease (life expectancy <1 year)	Relevant immunosuppressive therapies
Known severe brain injury	Systemic hematologic disorders
Age >75 years or age >70 plus ≥2 relative contraindications	Additional organ failure (except kidney)
End-stage lung disease	Frailty
Disseminated malignancy	Severe aortic regurgitation (VA ECMO)
Child-Pugh C liver cirrhosis	Severe peripheral vascular disease (VA ECMO)
<1 year after allogeneic stem cell transplantation	Chronic heart failure NYHA IV (without option for heart transplantation or ventricular assist device)

*Age limits possibly will have to be adapted according to the course of the pandemic

NYHA New York Heart Association

13. Wiedemann D, Bernardi MH, Distelmaier K, Goliasch G, Hengstenberg C, Hermann A, et al. Recommendations for extracorporeal membrane oxygenation (ECMO) in COVID-19 patients : Consensus paper of the Medical University of Vienna. Wien Klin Wochenschr. 2020;132(21-22):671-6.
14. Bartlett RH, Ogino MT, Brodie D, McMullan DM, Lorusso R, MacLaren G, et al. Initial ELSO Guidance Document: ECMO for COVID-19 Patients with Severe Cardiopulmonary Failure. ASAIO J. 2020;66(5):472-4.
15. Badulak J, Antonini MV, Stead CM, Shekerdemian L, Raman L, Paden ML, et al. Extracorporeal Membrane Oxygenation for COVID-19: Updated 2021 Guidelines from the Extracorporeal Life Support Organization. ASAIO J. 2021;67(5):485-95.

ESM table 2: List of COVID-19 intensive care units (ICUs) in Tyrol, Austria who participated in the Tyrolean COVID-19 Intensive Care Registry (Tyrol-CoV-ICU-Reg)

<table>
<tr><th></th><th></th></tr>
<tr><td>Medical University Innsbruck, Innsbruck, Austria</td><td>First wave:
Medical ICU, Department of Internal Medicine
Neurosurgical ICU, Department of Neurosurgery
Transplantation ICU, Department of General and Surgical Intensive Care Medicine/Department of Anesthesia and Critical Care Medicine
Recovery room “OZA” (adapted as temporary ICU), Department of General and Surgical Intensive Care Medicine/Department of Anesthesia and Critical Care Medicine
Recovery room “KHZ” (adapted as temporary ICU), Department of General and Surgical Intensive Care Medicine/Department of Anesthesia and Critical Care Medicine
Pediatric Intensive Care Unit, Department of Pediatrics
Second, third and fourth wave:
Medical ICU, Department of Internal Medicine
Neurosurgical ICU, Department of Neurosurgery
Transplantation ICU, Department of General and Surgical Intensive Care Medicine/Department of Anesthesia and Critical Care Medicine
General surgery ICU, Department of General and Surgical Intensive Care Medicine/Department of Anesthesia and Critical Care Medicine
Cardiac surgery ICU, Department of General and Surgical Intensive Care Medicine/Department of Anesthesia and Critical Care Medicine
Pediatric Intensive Care Unit, Department of Pediatrics</td></tr>
<tr><td>Hospital Hall, Hall, Austria</td><td>ICU, Department of Anesthesia and Intensive Care Medicine</td></tr>
<tr><td>Hospital Kufstein, Kufstein, Austria</td><td>ICU, Department of Anesthesia and Intensive Care Medicine</td></tr>
<tr><td>Hospital Lienz, Lienz, Austria</td><td>ICU, Department of Anesthesia and Intensive Care Medicine</td></tr>
<tr><td>Hospital Reutte, Reutte, Austria</td><td>ICU, Department of Anesthesia and Intensive Care Medicine</td></tr>
<tr><td>Hospital Schwaz, Schwaz, Austria</td><td>ICU, Department of Anesthesia and Critical Care Medicine</td></tr>
<tr><td>Hospital St. Johann i.T., St. Johann in Tyrol, Austria</td><td>ICU, Department of Anesthesia and Intensive Care Medicine</td></tr>
<tr><td>Hospital Zams</td><td>Medical ICU, Department of Internal Medicine

Surgical ICU, Department of Anesthesiology and Critical Care Medicine</td></tr>
</table>
